# Consensus statement on safety measures for pressurized intraperitoneal aerosol chemotherapy

**DOI:** 10.1515/pp-2021-0125

**Published:** 2021-11-02

**Authors:** Arnaud Girardot-Miglierina, Daniel Clerc, Mohammad Alyami, Laurent Villeneuve, Olivia Sgarbura, Marc-André Reymond, Martin Hübner, on behalf of the ISSPP PIPAC study group, Abba Julio, Afifi Adnane, Mortensen Michael Bau, Bharath G., Bhatt Aditi, Yan So Jimmy Bok, Brandl Andreas, Ceelen Wim, Cortes-Guiral Delia, Courvoiser Thomas, Coget Julien, de Hingh Ignace H., Delhorme Jean-Baptiste, Deo Suryanarayana S. V., di Giorgio Andrea, Dumont Frederic, Escayola Cecilia, Ezanno Anne-Cécile, Gagni`ere Johan, Galindo Julio, Glatz Torben, Jäger Tarkan, Jarra Maximilian, Katdare Ninad, Kepenekian Vahan, Khomyakov Vladimir M., Kothonidis Konstantinos, Laplace Nathalie, Lavoue Vincent, Lehmann Kuno, Lynch Craig, Mehta Sanket, Moldovan Bogdan, Nissan Aviram, Nowacki Maciej, Orry David, Ortega Pérez Gloria, Pabst Urs G., Paquette Brice, Paskonis Marius, Piso Pompiliu, Pocard Marc, Rau Beate, Reymond Marc, Ris Frederic, Robella Manuela, Silvestre-Rodriguez José, Singh Shivendra, Somashekhar S. P., Soravia Claudio, Sourrouille Isabelle, Taibi Abelkader, Tempfer Clemens, Torkington Jared, Vizzielli Giuseppe, Willaert Wouter

**Affiliations:** Department of Visceral Surgery, Lausanne University Hospital CHUV, University of Lausanne (UNIL), Lausanne, Switzerland; Department of General Surgery and Surgical Oncology, Oncology Center, King Khalid Hospital, Najran, Saudi Arabia; Department of Public Health, Clinical Research and Epidemiological Unit, Lyon University Hospital, Lyon, France; University of Lyon, Lyon, France; Department of Surgical Oncology, Cancer Institute Montpellier (ICM), Montpellier, France; University of Montpellier, Montpellier, France; IRCM, Institut de Recherche en Cancérologie de Montpellier, INSERM U1194, Université de Montpellier, Montpellier, France; Department of General and Transplant Surgery, University Hospital Tübingen and National Center for Pleura and Peritoneum, Tübingen, Germany; Digestive Surgery, University Hospital Grenoble Alpes, Grenoble, France; Surgical Oncology, Casablanca, Marocco; Department of Surgery, Odense Pancreas Center [OPAC], Odense University Hospital, Odense, Denmark; Department of Surgical Oncology, Malleswaram, Bangalore, India; Department of Surgical Oncology, Zydus Hospital,Ahmedabad, India; Department of Surgery, National University of Singapore, Yong Loo Lin School of Medicine, Singapore, Singapore; Digestive Unit, Champalimaud Foundation, Lisbon, Portugal; Department of GI Surgery and Cancer Research Institute Ghent [CRIG], Ghent University Hospital, Ghent, Belgium; Department of Colorectal Surgery, King Khalid Hospital, Nejran, Saudi Arabia; Department of Digestive Surgery, CHU Poitiers, Poitiers, France; Department of Digestive Surgery, CHU Rouen, Rouen, France; Department of Surgery, Catharina Hospital, Eindhoven, the Netherlands; Department of Digestive Surgery, CHU Hautepierre, Strasbourg, France; Department of Surgical Oncology, Dr BRA IRCH, AIIMS, New Delhi 110029, India; Department of Digestive Surgery, Fondazione Policlinico Universitario Agostino Gemelli IRCCS, Rome, Italy; Department of Surgical Oncology, Institut Canc´erologique de l’Ouest, Saint Herblain, France; Division of Gynaecologic Surgery, Clinica del Pilar, Barcelona, Spain; Department of Surgery,HIA Begin, Saint Mand´e, France; Department of Hepatobiliary and digestive surgery, CHUEstaing, Clermont-Ferrand, France; Department of General and Digestive Surgery, Hospital Universitario Ramón y Cajal, Madrid, Spain; Department of General and Visceral Surgery, Medical Center – University of Freiburg, Freiburg, Germany; Department of Surgery, Paracelsus Medical University, Salzburg, Austria; Department of General Surgery, Campus Virchow Klinikum, Charit´e, Universitätsmedizin Berlin, Berlin, Germany; Department of General Oncology, Sir H. N. Reliance Foundation Hospital and Research Centre, Mumbai, India; Department of Digestive Surgery, Centre Hospitalier Lyon Sud, Lyon, France; Department of thoracoabdominal cancer surgery, P.A. Hertsen Moscow Oncology Research Center, Moscow, Russia; Department of Digestive Surgery, CHR Val de Sambre, Sambreville, Belgium; Department of Digestive Surgery, Centre Hospitalier Lyon Sud, Lyon, France; Department of Gynecology, CHU Rennes, Rennes, France; Department of Surgery and Transplantation, Univer sity Hospital of Zurich, Zurich, Switzerland; Division of Cancer Surgery, Peter MacCallum Cancer Centre, Melbourne, Victoria, Australia; Depart ment of Surgical Oncology, Saifee Hospital, Mumbai, India; Department of General Surgery, “Sf. Constantin” Private Hospital Braşov, Romania; Department of General and Oncological Surgery- Surgery C, The Chaim Sheba Medical Center, Tel Hashomer, Ramat Gan, Israel; Department of Surgical Girardot-Miglierina et al.: PIPAC safety consensus 147 Oncology, Ludwik Rydygier’s Collegium Medicum, Nicolaus Copernicus University in Torun, Bydgoszcz, Poland; Department of Surgical Oncology, Centre Geor ges-François Leclerc, Dijon, France; Department of Surgical Oncology, MD Anderson, Madrid, Spain; Department of Surgery, RuhrUniversity Bochum, Bochum, Germany; Department of Digestive Surgery, CHU Jean Minjoz, Besançon, France; Centre of Abdominal Surgery, Vilnius University Hospital SantariškiųKlinikos,Vilnius, Lithuania; Department of General and Visceral Surgery, Barmherzige Brueder Hospital Regensburg, Regensburg, Germany; Department of Digestive and Visceral Surgery, APHP Lariboisi`ere, Paris; Department of Surgery, Campus Virchow-Klinikum and Charit´e Campus Mitte, Charit´e-Universitätsmedizin Ber lin, Germany; Department of Surgery, University of Tübingen, Tübingen, Germany; Division of Digestive Surgery, University Hospitals of Geneva, Geneva, Switzerland; Unit of Surgical Oncology, Candiolo Cancer Institute-FPO, IRCCS, Turin, Italy; Department of General Surgery, Hospital Universitario de Gran Canaria Dr. Negrin, Las Palmas de Gran Canaria, Spain; Department of Gastro-intestinal Oncosurgery & Liver Transplantation, Rajiv Gandhi Cancer Institute, New Delhi, India; Department of Surgical Oncology, Manipal Comprehensive Cancer Center, Manipal Hospital, Bangalore, India; Laparoscopic Robotic Surgery, Clinique G´en´erale-Beaulieu, Geneva, Switzerland; Department of Surgical Oncology, Institut Gustave Roussy, Villejuif, France; Department of Digestive Surgery, CHU Dupuyren, Limo ges, France; Department of Obstet rics and Gynecology, Ruhr-Universität Bochum, Bochum, Germany; Department of General Surgery, University Hospital of Wales, Cardiff, United Kingdom; Division of Gynecologic Oncology, Catholic University of the Sacred Heart, Rome, Italy; Department of GI Surgery and Cancer Research Institute Ghent [CRIG], Ghent University Hospital, Ghent, Belgium.

**Keywords:** education and training, expert consensus, personal protective equipment, PIPAC, safety

## Abstract

**Objectives:**

Pressurized intraperitoneal aerosol chemotherapy (PIPAC) is a promising treatment for peritoneal cancer that entails, however, potential risks for the caregivers in the operating room (OR). This study aimed to reach a consensus within the PIPAC community on a comprehensive safety protocol.

**Methods:**

Active PIPAC centers were invited to participate in a two-round Delphi process on 43 predefined items: concise summaries of the existing evidence were presented together with questions formulated using the population, intervention, comparator, and outcome framework. According to the Grading of Recommendations Assessment, Development, and Evaluation, the strength of recommendation was voted by panelists, accepting a consensus threshold of ≥50% of the agreement for any of the four grading options, or ≥70% in either direction.

**Results:**

Forty-seven out of 66 invited panelists answered both rounds (response rate 76%). The consensus was reached for 41 out of 43 items (95.3%). Strong and weak recommendations were issued for 30 and 10 items, respectively. A positive consensual recommendation was issued to activate laminar airflow without specific strength, neither strong nor weak. No consensus was reached for systematic glove change for caregivers with a high risk of exposure and filtering facepiece mask class 3 for caregivers with low risk of exposure.

**Conclusions:**

A high degree of consensus was reached for a comprehensive safety protocol for PIPAC, adapted to the risk of exposure for the different caregivers in the OR. This consensus can serve as a basis for education and help reach a high degree of adherence in daily practice.

## Introduction

Pressurized intraperitoneal aerosol chemotherapy (PIPAC) has been developed as a new drug delivery system to treat patients with nonresectable peritoneal metastases of various primaries [1], [2], [3].

Chemotherapeutic agents (CA) are manipulated during PIPAC. Thus, there is a potential risk of exposure to liquid and aerosolized CA for caregivers present in the operating room (OR) during the procedure [4]. Before the first-in-human use, and in collaboration with an independent organization certified for occupational health risk assessment (DEKRA Industrials GmbH, Stuttgart, Germany), the pioneer team in Bielefeld performed a detailed risk assessment and developed a dedicated safety protocol. This original safety protocol included tightness of the abdomen, laminar airflow ventilation in the OR, controlled aerosol waste, remote control of the procedure, and wearing protective clothing, gloves, and glasses. Simulations of the worst-case scenario (total release of the chemotherapeutic aerosol into the OR with the person’s presence for 30 min) calculated an inhaled CA dose between 1:100,000 and 1:1,000,000 of a usual chemotherapeutic dose [5]. The safety protocol was successfully validated under clinical conditions during the first PIPAC procedures with no platin traces detected in the air (detection limit: 0.000009 mg/m^3^). Assuming a platin exposition of 8 h daily, a maximal substance index <18% of the acceptable exposition limit was found, allowing the audit to conclude that the implemented protective measures were meeting the regulatory requirements in Germany (Technische Regeln für Gefahrstoffe [TRGS] 402).

In the following years, multiple PIPAC safety audits were conducted in several European countries [4], [5], [6], [7], [8], [9], [10], [11], [12]. In most audits, PIPAC was performed in OR with advanced ventilation OR system meeting the norm ISO 14644-1 class ≤5 but no laminar airflow. In none of these studies, traces of platin were detected in the air [4, 6, 7, 9, 11, 13, 14]. However, various degree of contamination of instruments and surfaces was documented [6, 7, 9, 14, 15]. Assuming thorough implementation of the safety protocols, all independent audits concluded so far that PIPAC can be performed safely, meeting European and national legal and regulatory requirements. Biological monitoring in the blood [7, 16] or urine [8] of healthcare workers showed no traces of chemotherapeutic drugs after PIPAC. The German pioneer group implemented a Critical Incident Reporting System for the first 650 PIPAC procedures. Two minor incidents related to disconnection in the tubing system were reported. No severe incident, in particular no leakage of the toxic aerosol, was recorded [5].

In the meantime, PIPAC is diffusing into clinical practice worldwide. Participation in an International Society for the Study of Pleura and Peritoneum (ISSPP) PIPAC training course is required for technology access, and the safety protocols are an essential component of this course [17]. However, a recent survey amongst PIPAC expert centers worldwide showed variable adherence to protective measures [18]. For example, many PIPAC centers now recommend using filtering facepiece (FFP)-2 masks, which were not originally considered necessary by the DEKRA organization. Another recent study on everyday practice and the need for information relating to the risk of exposure suggested that adherence to different protective measures was variable but that the need for continuous education was high [19]. Furthermore, knowledge concerning the risks of CA and the safety protocol was variable amongst OR professionals, including surgeons, anesthetists, anesthesia nurses, scrub nurses, and cleaning staff.

Thus, there is a need for establishing a consensus on the required PIPAC safety measures. Therefore, we designed a Delphi study to reach an agreement on a comprehensive safety protocol among active PIPAC centers worldwide.

## Materials and methods

The present study methodology consisted of a two-round Delphi consensus process and was developed in agreement with current standards for developing consensus guidelines [20]. The project was initiated in early 2020 following two surveys on current PIPAC safety practices and perception [18, 19]. The study was performed under the precepts established by the Declaration of Helsinki.

The authors of this study formed the Guidelines development group (GDG), which consisted of internationally represented surgeons with extensive expertise with PIPAC therapy and developing consensus guidelines. The pioneer German team contributed with its 10-year expertise in occupational health protocols for PIPAC. Four GDG members (MA, LV, OS, and MH) are an active part of the educational group of the ISSPP and are in charge of the training curriculum for PIPAC, including its safety aspects [17].

**Figure 1:**
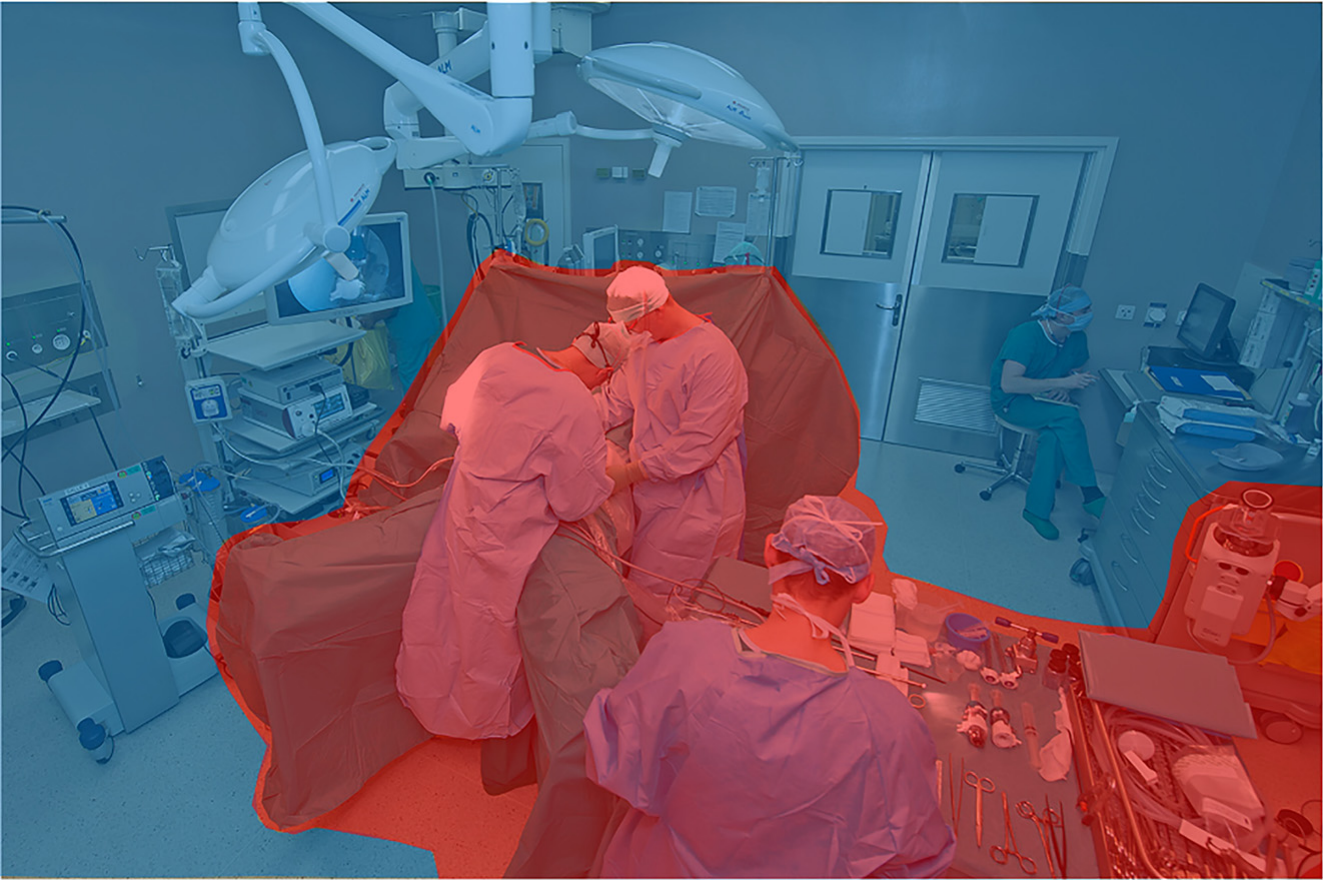
PIPAC procedure operating room scenario. Definition of high risk vs. low risk for caregivers during PIPAC procedure. Caregivers at high risk of potential direct exposure to CA (red zone) include surgeons and the surgical team (scrub nurses and surgical assistant), and personnel assigned to the manipulation of the injector. Caregivers at low risk of potential direct exposure to CA (blue zone) include the anesthesiology team, circulators, visitors, and the cleaning staff. Of note, during the remote administration of aerosol chemotherapy and until pneumoperitoneum evacuation, any caregivers entering the operating room are considered at high risk of exposure to aerosolized chemotherapy.

Delphi questions taking into account all different aspects of the topic were defined *a priori* and formulated according to the population, intervention, comparator, and outcome framework [21], by five members of the GDG (AGM, DC, MA, OS, and MH). For determining the questions, the existing literature was analyzed, looking for the best available evidence from the first description of the PIPAC procedure [3] up to December 2019. Questions were divided into four broad categories: i) personal protective equipment (PPE), ii) environmental protection, iii) prevention of exposure to aerosolized chemotherapy, and iv) general preventive measures. Of note, these questions included items that have not been specified or explicitly mentioned in prior works on occupational health aspects of PIPAC [7], [8], [9]. Furthermore, personal protective measures were studied separately for caregivers at high or low risk of exposure, respectively. The definition of caregivers at high or low risk of exposure is outlined in Figure 1 and further defined in the Supplementary Material.

The targeted expert panel included nonselected leaders of all active PIPAC programs identified before December 2019 and previously invited for participation in the development of consensus guidelines for PIPAC technical aspects [22]. No center was deliberately excluded from the process.

An interactive online survey (SurveyMonkey Inc., San Mateo, CA) was sent to the expert panel. Delphi questions were presented together with concise summaries and references to the evidence (Supplementary Material). Experts were asked to provide their level of agreement for, or against, the use of each detailed safety measure and give closed-end recommendations on each item, by the use of a two-sided scale (strong positive, weak positive, weak negative, and strong negative), according to the Grading of Recommendations Assessment, Development, and Evaluation approach [23]. All responders of Delphi’s first round were invited to participate in the second round. The second Delphi round presented the same information and questions with additional feedback on the results of the first round. Every participant had one month to answer the survey, and nonresponders received a minimum of three reminders.

### Statistical analysis

The GDG analyzed the data. Descriptive statistics were used to summarize the results of the expert consensus. The consensus was defined as ≥50% of the agreement for any of the four grading options, or as 70% agreement for a combined weak or strong recommendation, regardless of the direction, negative or positive.

## Results

There were 66 participants. Response rates for Delphi’s first round were 52 (87%), and 47 of these responders completed then Delphi’s second round, resulting in a final response rate of 76%. Surgeons represented 46 of the responders completing the entire Delphi, holding a consultant position for 31 (67%).

The consensus was reached for 41 out of 43 items (95.3%). In summary, 26 recommendations (60.4%) were strong positive, while the remainders were either weak positive (n=9, 20.9%), weak negative (n=1), or strong negative (n=4, 9.3%). One recommendation (activation of laminar airflow) reached consensus with >70% of combined strong and weak positive agreement. No consensus was reached for two items (4.7%) after the two Delphi rounds, namely the change of gloves after 30 min for caregivers with a high risk of exposure and the use of FFP mask class 3 for caregivers with a low risk of exposure.

The following paragraphs provide the synopsis of evidence and degree of consensus divided into four categories of safety measures: (I) PPE, (II) environmental protection, (III) prevention of exposure to aerosolized chemotherapy, and (IV) general preventive measures. Details are provided in Supplementary Material, Appendix 1.

### Personal protective equipment (PPE)

**Figure 2:**
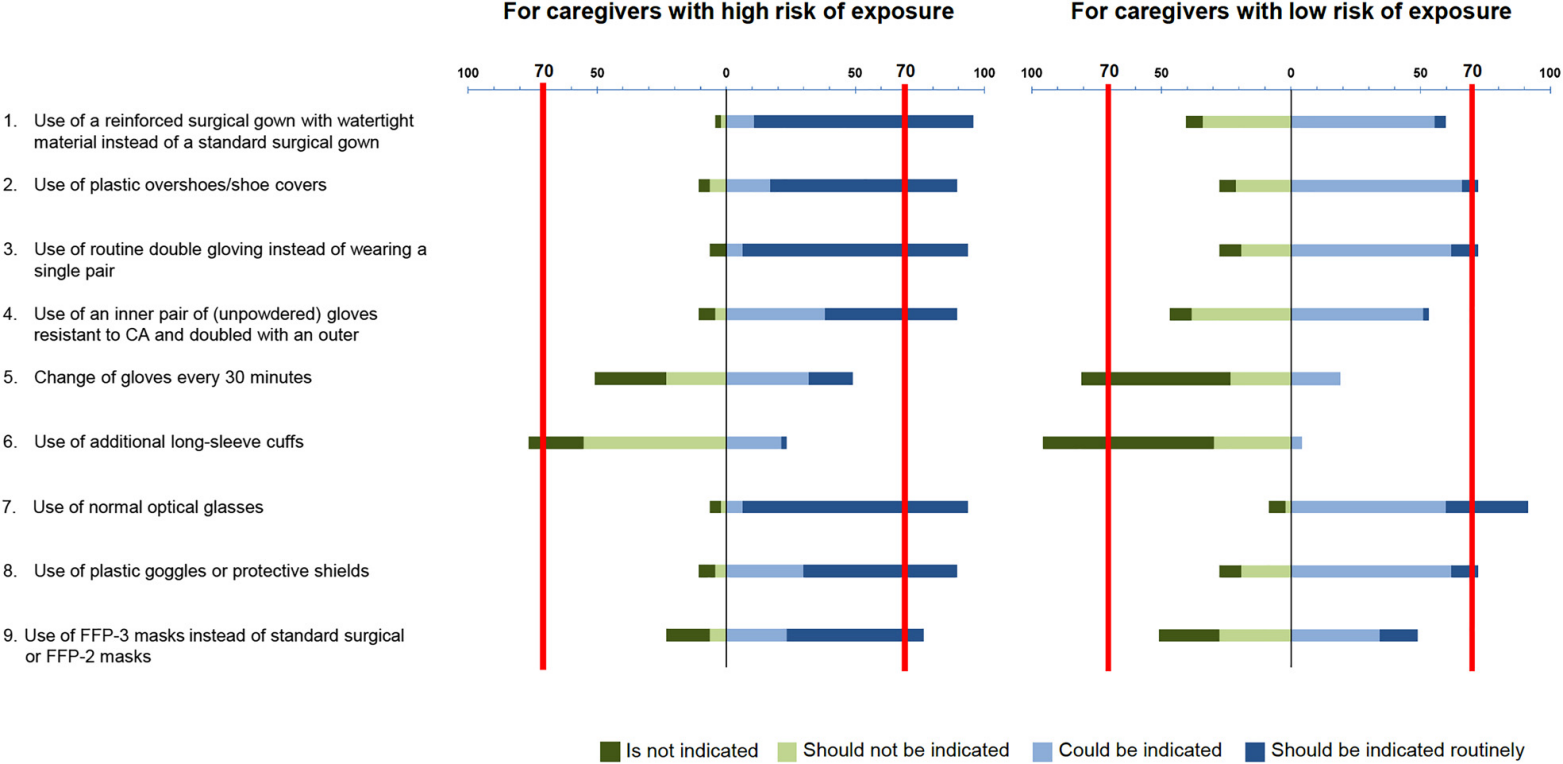
Consensus for PPE for the prevention of exposure to liquid or aerosolized chemotherapy agents.

**Figure 3:**
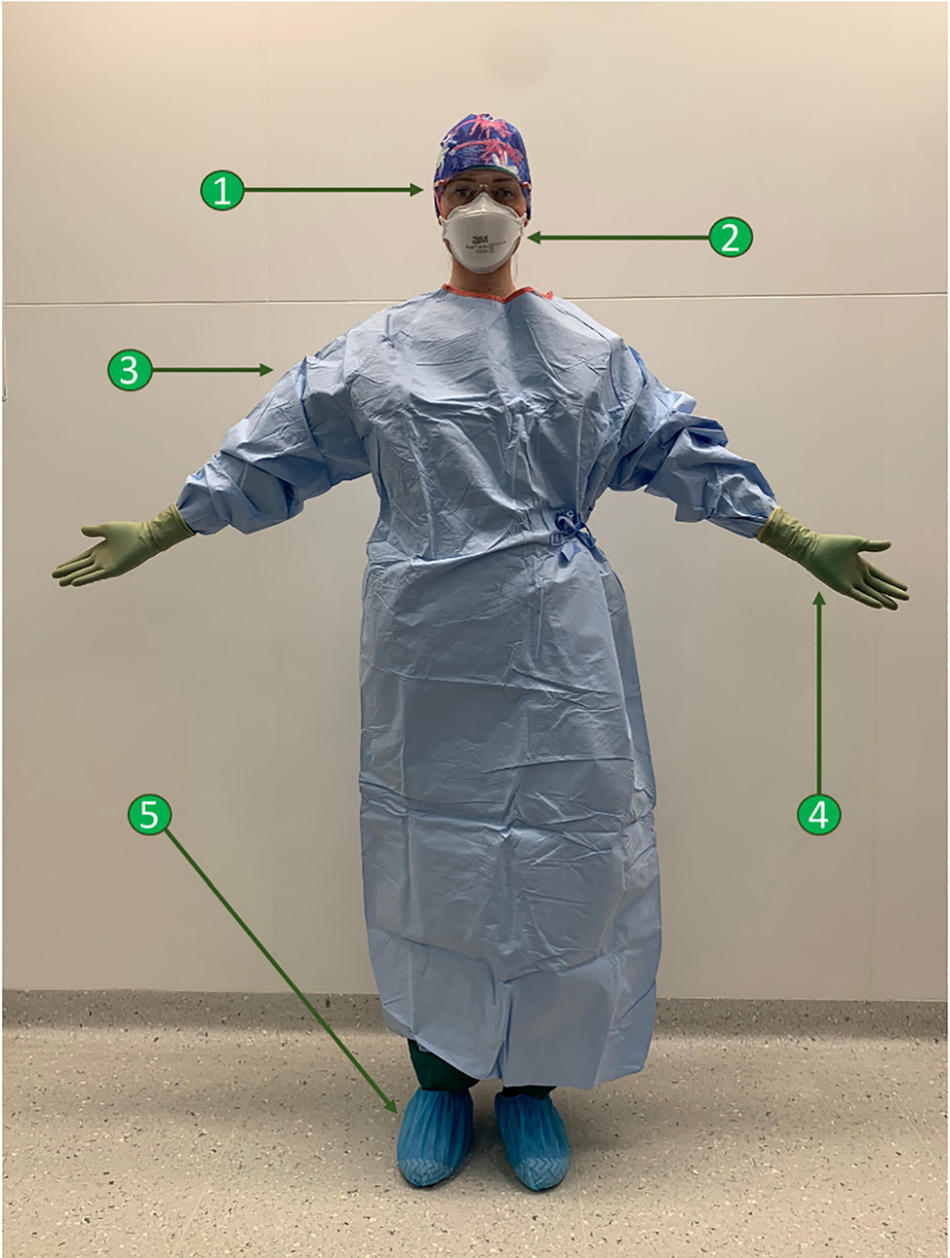
Recommended PPE during PIPAC procedure for caregivers at high risk of exposure. Optimal PPE for caregivers at high risk of exposure during PIPAC: (1) Specific ocular protection (plastic goggles or eye shields), (2) FFP mask class 3, (3) reinforced surgical gown, (4) double gloving with an inner pair resistant to chemotherapy agents, and (5) plastic overshoes.

Exposure to CA during PIPAC can occur through direct contact (dermal or ocular) with contaminated surfaces or materials [4]. Several studies examined gloves, hands, devices, injectors, trocars, and floor wiping samples for platin traces and reported highly variable contamination levels [6, 8, 9]. Differences in current practices regarding the PPE required for performing PIPAC have been observed among expert centers [18]. In this Delphi study, there was a large consensus for the PPE needed to perform PIPAC safely. Figure 2 shows the results stratified by the risk of exposure. No agreement was reached for changing gloves every 30 min (high risk of exposure) instead of keeping the same gloves from the beginning to the end of the procedure. Optimal PPE for caregivers at increased risk of exposure during the PIPAC procedure is summarized in Figure 3. However, there is no transdermal absorption of platin or anthracyclins, and the risk linked to exposition to liquids is probably limited to local cutaneous or ocular toxicity. Biological monitoring studies confirmed the efficacy of PPE: no platin traces were found in blood and urine samples of persons performing PIPAC regularly [8, 9], even after 1,200 procedures [16].

### Environmental protection

**Figure 4:**
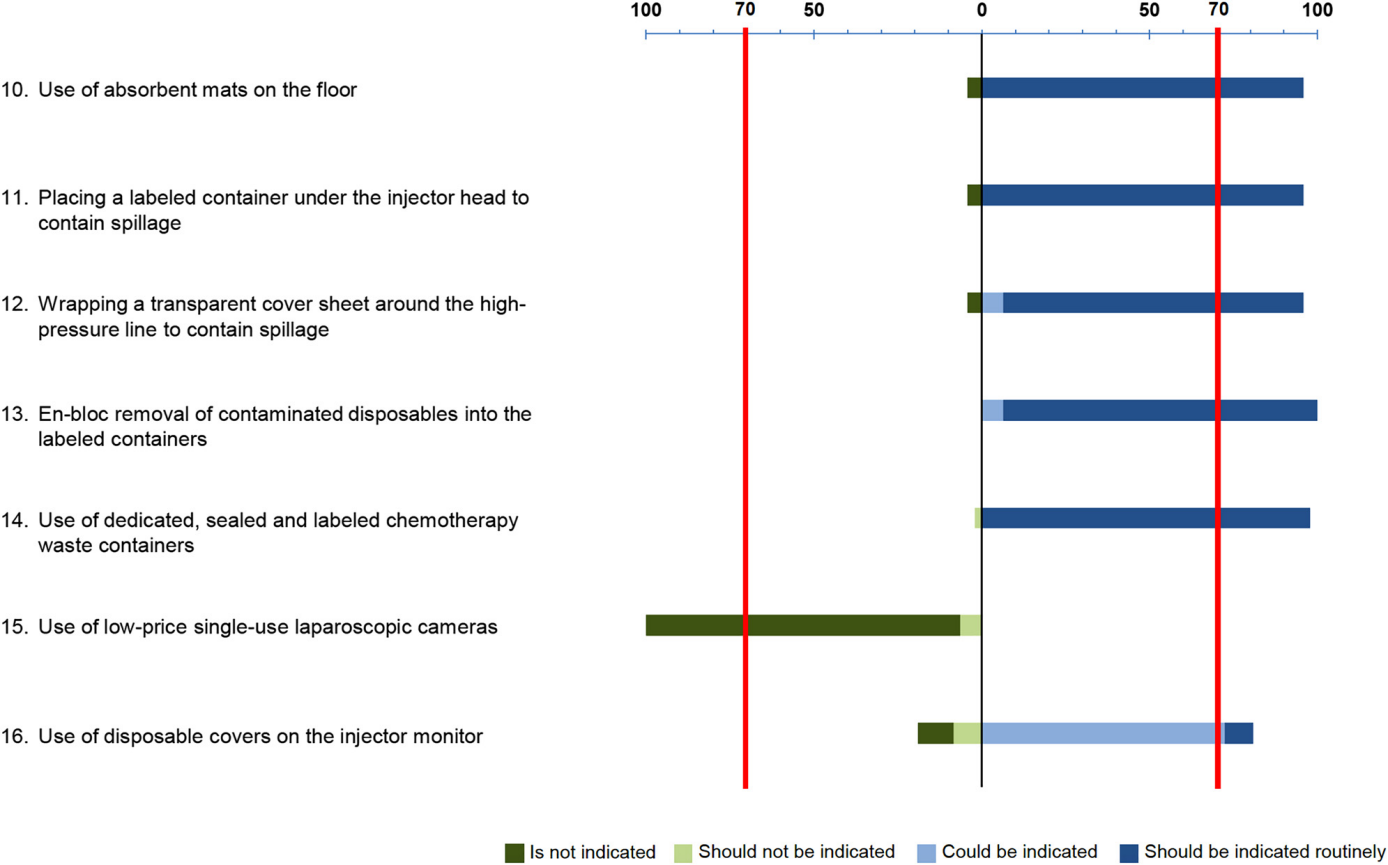
Consensus for environmental protection.

The degree of consensus for the different measures for environmental protection is detailed in Figure 4. Among the seven items evaluated regarding environmental protection, a strong positive recommendation was found in five, and their use should be routinely indicated: absorbent mats (95.7% of agreement), labeled container under the injector head (95.7%), transparent cover sheet (89.4%), “en bloc” removal (93.6%), and dedicated labeled waste containers (97.9%). The use of a disposable cover over the injector monitor met a weak positive recommendation (72.3%) and could be indicated. Strong negative recommendation (93.6%) was found for the use of single-use laparoscopic camera and is therefore not indicated.

The original PIPAC safety protocol [4] recommends positioning a watertight drape on the floor and placing a waste bin for chemicals beneath the angioinjector head. Disposable covers on the injector monitor during PIPAC could help prevent the transfer of potential contamination to other surfaces [6]. After line disconnection incidents between the syringe and the high-pressure line, an additional recommendation was to protect the high-pressure line with a sterile plastic bag [16]. The aerosolizer, the line, and the syringe must be disposed of as a whole. One multi-center study [6] demonstrated a decrease in local contamination when surgical disposables were removed “en bloc” compared to removal after disconnection [24]. In order not to endanger third parties unnecessarily, every single-use material must be collected immediately by the surgical staff (wearing PPE) in specially designed and labeled waste containers. This waste material includes, among other things: empties (syringes, infusion containers, and lines), single-use instruments (e.g., trocars, aerosolizing device, etc.), operating drapes and gauzes, stitches, and needles. The watertight waste containers should be labeled in accordance with the dangerous goods and waste legislation, stating the nature of the waste – “cytotoxic and cytostatic waste”, the UN number under dangerous goods law, and the hospital (sender) address. The hermetically closed containers are to be hand over undamaged to the disposal company. The laparoscopic camera (precisely: the Hopkins optics) is the only multi-use instrument exposed to CA. One study [12] demonstrated minimal traces of platinum on 1/3 Hopkins optics directly after PIPAC, but not after the sterilization process. This shows that laparoscopic cameras can be safely reused after PIPAC.

### Prevention of exposure to aerosolized CA

**Figure 5:**
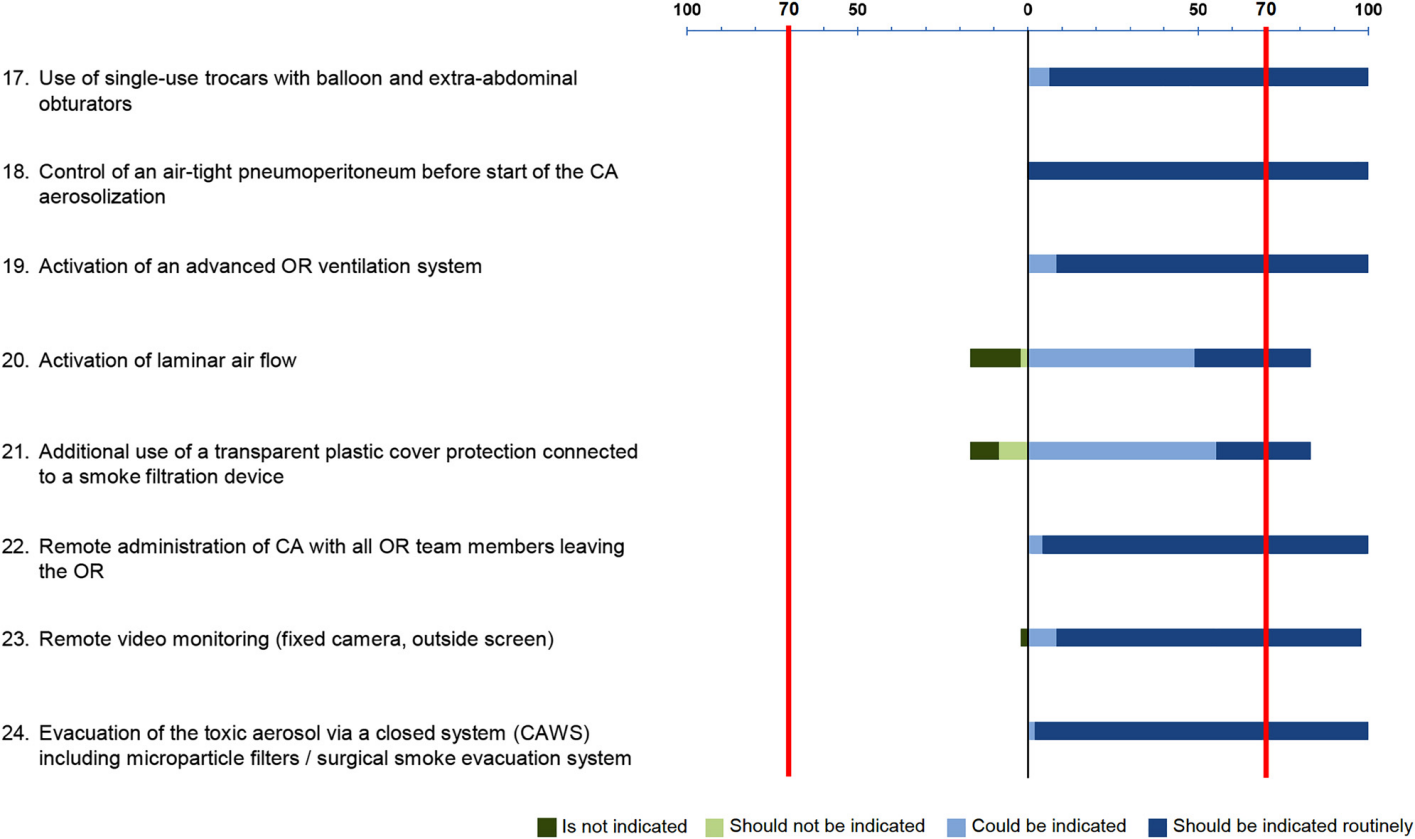
Consensus for prevention of exposure to aerosolized chemotherapy agents.

The degree of consensus for the different measures for preventing exposure to aerosolized CA is given in Figure 5. Eight items evaluated the prevention of exposure to aerosolized CA. The strong positive recommendation was found in six and should be routinely indicated: use of disposable balloon trocars (93.6% of agreement), airtight pneumoperitoneum (100%), advanced OR ventilation system (91.5%), remote chemotherapy administration (95.7%), remote video monitoring of aerosolization (89.4%), and safe toxic aerosol evacuation (97.9%). Two remaining items met weak positive recommendation, and therefore could be indicated: laminar airflow activation (48.9%), and additional plastic cover protection with smoke filtration (“French system”) (55.3%).

A hazard specific to PIPAC is a potential inhalation exposure caused by possible CA leakage during administration [4]. A number of studies have investigated OR air contamination with cisplatin by measuring its concentration in air samples: none of the measurements performed in Germany (Bielefeld [4], Herne [11], Tübingen [6], Regensburg [6], and Leipzig [15]), France (Lyon, Strasbourg [14]), Belgium (Gent [9]), Denmark (Odense [7]), and The Netherlands (Eindhoven [25]) showed traces of platin in the air. The field study of Ametbischler et al., conducted under routine conditions at two different centers (Tübingen and Regensburg), is quantitatively (14 PIPAC procedures) and qualitatively (platin detection limit of 0.000000000003 g platin in 1,000 L air). Together, all these studies provide solid evidence that the three-level confinement system implemented for PIPAC effectively prevents inhalation exposure to toxic aerosols.

### General preventive measures

**Figure 6:**
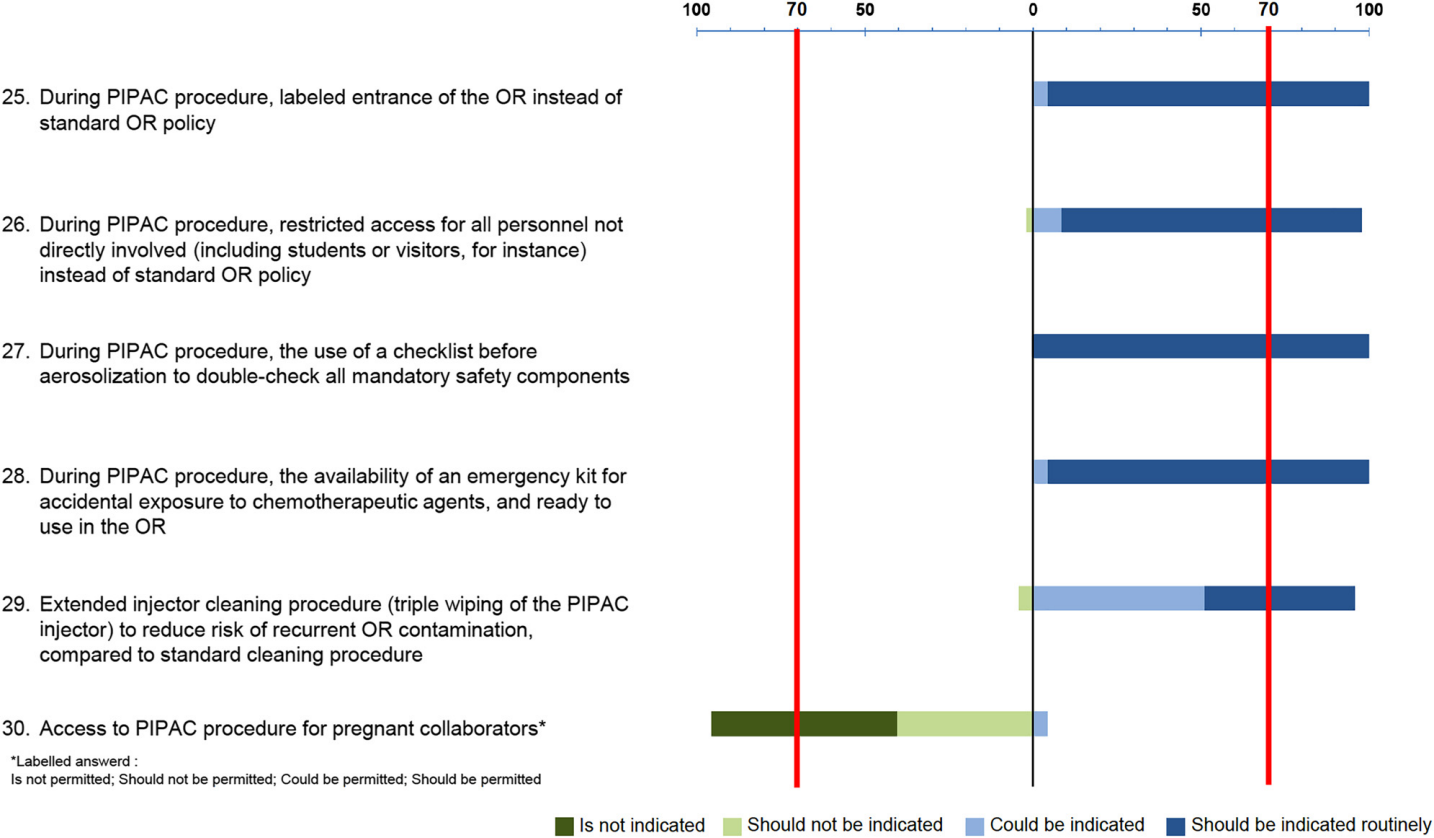
Consensus for general preventive measures.

High degree of consensus was reached for the use of general preventive measures (Figure 6). Access to the OR during PIPAC procedures is restricted in most centers, and the OR is usually labeled as a hazard area. The original PIPAC safety checklist [4] is widely adopted, with some minor local adaptations. This safety list is advocated in ISSPP training modules [22]. Emergency kits are broadly available, including absorbent devices, mild soap, bleach, and eyewash kit, for quick CA absorption and first medical aid [26]. Pregnant women should not participate in PIPAC procedures, in analogy to hyperthermic intraperitoneal chemotherapy (HIPEC) procedures [26, 27]. Platinum contamination on the floor of the OR has been detected up to 3 days after the HIPEC procedure, underlining the importance of effective cleaning methods [28]. In contrast, floor contamination is barely observed after PIPAC. However, a significant, in some cases, high surface contamination of the angio-injector has been documented after PIPAC, suggesting leakage during syringe manipulation and connection, as well as insufficient cleaning methods. In one study [6], contamination was higher before PIPAC as compared to after PIPAC, while another study [8] showed that contamination remained after cleaning. These results imply that the cleaning of the angioinjector has received insufficient attention so far. A revised cleaning method with triple-wiping was reported to reduce injector contamination [8].

**Table 1:** Consensus on the need of information and training.

**Figure:**
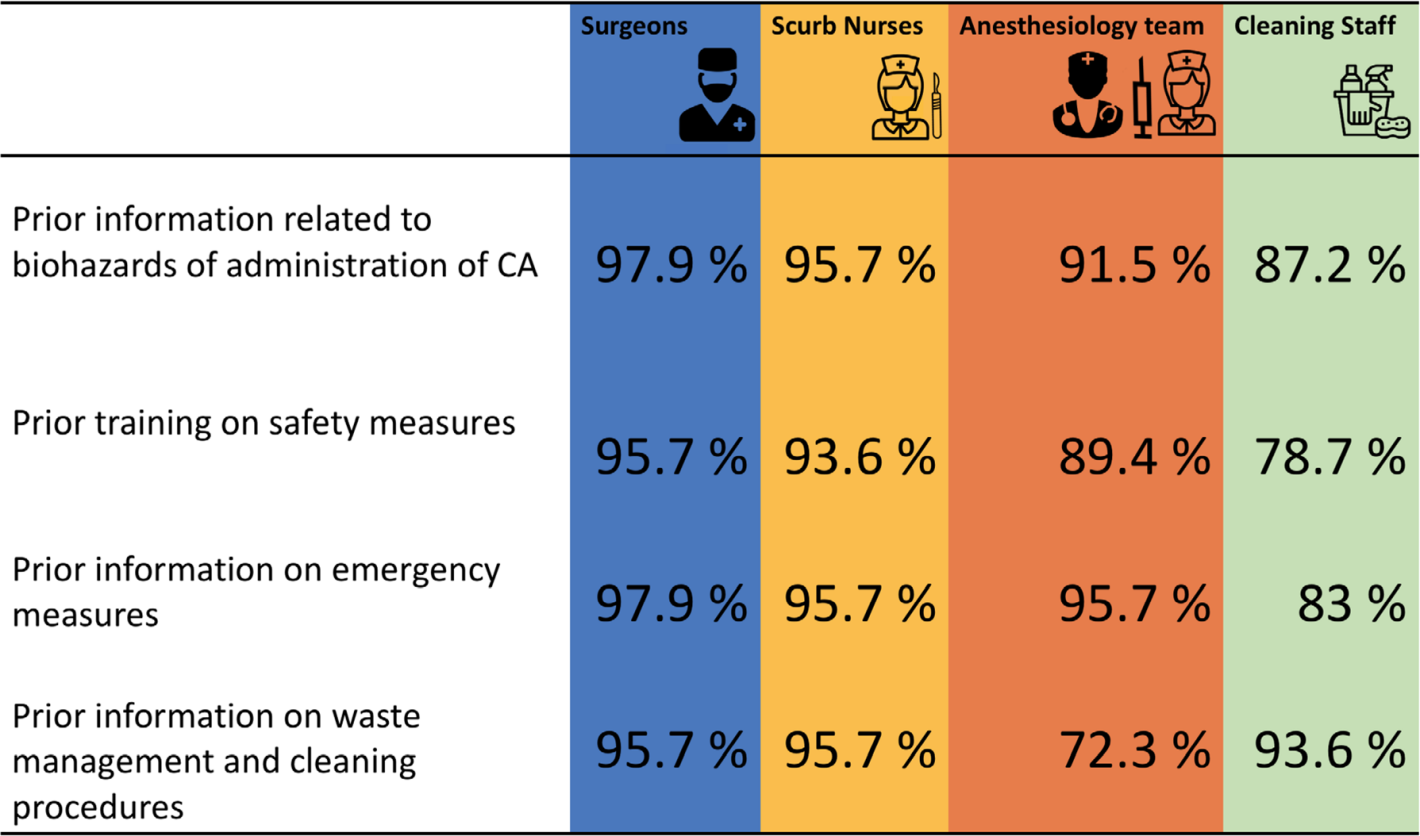

Data outlined as percentage of strong positive recommendation.

In this Delphi study, the need for information and training was estimated to be high (Table 1). A recent survey amongst PIPAC expert centers showed a rather low rate of adhesion to protection measures. Safety awareness did not reach expected levels for a highly standardized procedure, with a lower information score among anesthesiologists and cleaning staff. Availability of emergency kits in case of accidental exposure was unknown for 50% of responders. Most OR team members seek supplementary information about the risks related to CA administration [18]. Another recent study demonstrated that nonmedical caregivers in the OR are aware of the occupational hazards related to the use of CA. However, there is a high need for continuous education for the healthcare personnel participating in PIPAC procedures [19].

## Discussion

In this Delphi study, a high degree of consensus was reached for a comprehensive safety protocol for PIPAC, adapted to the risk of exposure for the different caregivers in the OR.

Little is known about incidents or safety breaches during PIPAC procedures. All existing safety protocols have been adapted from the original protocol from the pioneer team in Germany which had been developed under the auspices of German regulatory bodies with a strong focus on healthcare safety. Early experience from Lausanne [22], reported only minor events during aerosolization, i.e. automatic stop of the injector due to pressure limitation and minor chemotherapy leaks, entirely confined by the cover sheet systematically used. Direct incidental exposure of the OR co-workers, has not been described [10]. In a recent survey assessing safety perception of intraperitoneal chemotherapy administration (PIPAC and HIPEC) from 211 OR co-workers [18], 28% reported being aware of at least one incident, without further details. Prospective multicentric auditing of safety breaches during PIPAC procedure might provide more detailed information on the subject in the future.

However, over the years, safety protocols have been slightly adapted, most likely due to physician preference on certain aspects and safety material availability, leading to some variations between different countries. In our study was not possible to reach a sufficient degree of consensus for the following measures:

### Laminar airflow

Although laminar airflow was considered mandatory in the initial risk evaluation, several independent studies showed that laminar airflow is not needed. An advanced OR ventilation system meeting the norm ISO 14644-1 class ≤5 is sufficient to prevent inhalation exposition during PIPAC [4, 6, 7, 9, 11, 15]. The French PIPAC centers developed an alternative protocol using a plastic drape covering the patient and tubing connected to a mobile HEPA filtering device [15]. This protocol was audited successfully by the national safety authority in France (INRS – *Institut National de Recherche et Sécurité*) [8, 29]. The alternative “French” system is currently widely used in France and other countries. In a study on two PIPAC procedures, no platin traces were measured in the air with a sensitivity of <0.00000002 g/m^3^ [14]. In spite of the supportive evidence available for this system, the German Worker’s insurance does not allow PIPAC performance in OR with no advanced ventilation/filtration system.

### FFP masks

Wearing FFP mask class 2 (filtering 94% of particles with a diameter between 0.01 and 1 μm) or class 3 (filtering 99% of particles) offers additional protection against inhalation exposition to toxic aerosols during PIPAC. On the other side, such masks were not recommended in the initial risk evaluation, assuming the OR ventilation/filtration system could reduce the inhalation risk to an acceptable minimal level. Numerous negative air measurements (see above) confirmed that the person’s safety during PIPAC is not dependent on wearing FFP class 2 or 3 masks. On the other side, the German workers’ insurance recommends wearing FFP-3 masks during HIPEC [30] and, in analogy, it appears reasonable to propose FFP class 2 or 3 masks as an additional protective measure.

### Surface contamination

The evidence available shows that there can be surface contamination (in particular of the angio-injector) after PIPAC. An important lesson from Ametbischler’s field study [6] is that the surface contamination varies by four orders of magnitude (=10.000 times) between individual PIPAC procedures. In some PIPAC procedures, surface contamination is absent after careful handling of the chemotherapy syringes and qualified instrument operation. In other procedures, significant contamination was detected, in some cases even high levels of contamination, probably following unqualified handling. These findings underline

– The need for adequate, repeated, documented training of the persons operating the angio-injector during PIPAC, in order to guarantee proper handling. It is legally and medically inacceptable to allow unqualified personal to operate the angioinjector.
– The need for adequate cleaning of the angioinjector after the procedure, through qualified personal wearing proper PPE.

There might be methodological issues with some studies, showing e.g. surface contamination before but not after PIPAC, or floor contamination in spite of the presence of a drape on the floor and with no air contaminations. Such findings suggest that chemotherapy traces were present before PIPAC, e.g. when PIPAC is performed in a room regularly used for HIPEC procedures handling larger volumes of chemotherapeutic solutions.

Taken together, this Delphi study shows a large consensus between PIPAC centers on most safety measures during PIPAC. Some practices differ, such as the use of a plastic cover drape with a mobile HEPA filtering system, or wearing FFP masks. This Delphi study is meant to support hospital management, physicians, nurses, and regulatory authorities in making decisions concerning appropriate safety measures during PIPAC. The systematic statement developed in this study reflect the current opinion of surgeons, anesthesia, and nurses on safety measures needed during PIPAC procedures, and do not reflect the opinion of the ISSPP.

The EC guidelines, in particular Directive 89/391/EEC, lay down the main principles to encourage improvement in the safety and health of workers at work. These principles are precised in further directives, e.g. 2004/37/EC “carcinogens or mutagens at work” and 2019/1831 “indicative occupational exposure limit values”. These directives have been translated into national laws and regulations, which might differ slightly between countries, and also between the EC and other locations in the world. In the EC, it is the responsibility of the employers to take the measures required by these laws and regulations.

In summary, a high degree of consensus was reached for a comprehensive and risk-adapted safety protocol for PIPAC for the different caregivers in the OR. This consensus can be a common basis for education and implementation and provide valuable guidance helping to reach high adherence and a safe procedure.

## Supplementary Material

Supplementary Material Details

## Appendix

### ISSPP PIPAC study group

Julio Abba (Digestive Surgery, University Hospital Grenoble Alpes, Grenoble, France); Adnane Afifi (Surgical Oncology, Casablanca, Marocco); Michael Bau Mortensen (Department of Surgery, Odense Pancreas Center [OPAC], Odense University Hospital, Odense, Denmark); G. Bharath (Department of Surgical Oncology, Malleswaram, Bangalore, India); Aditi Bhatt (Department of Surgical Oncology, Zydus Hospital, Ahmedabad, India); Jimmy Bok Yan So (Department of Surgery, National University of Singapore, Yong Loo Lin School of Medicine, Singapore, Singapore); Andreas Brandl (Digestive Unit, Champalimaud Foundation, Lisbon, Portugal); Wim Ceelen (Department of GI Surgery and Cancer Research Institute Ghent [CRIG], Ghent University Hospital, Ghent, Belgium); Delia Cortes-Guiral (Department of Colorectal Surgery, King Khalid Hospital, Nejran, Saudi Arabia); Thomas Courvoiser (Department of Digestive Surgery, CHU Poitiers, Poitiers, France); Julien Coget (Department of Digestive Surgery, CHU Rouen, Rouen, France); Ignace H. de Hingh (Department of Surgery, Catharina Hospital, Eindhoven, the Netherlands); Jean-Baptiste Delhorme (Department of Digestive Surgery, CHU Hautepierre, Strasbourg, France); Suryanarayana S. V. Deo (Department of Surgical Oncology, Dr BRA IRCH, AIIMS, New Delhi 110029, India); Andrea di Giorgio (Department of Digestive Surgery, Fondazione Policlinico Universitario Agostino Gemelli IRCCS, Rome, Italy); Frederic Dumont (Department of Surgical Oncology, Institut Cancérologique de l’Ouest, Saint Herblain, France); Cecilia Escayola (Division of Gynaecologic Surgery, Clinica del Pilar, Barcelona, Spain); Anne-Cécile Ezanno (Department of Surgery, HIA Begin, Saint Mandé, France); Johan Gagnière (Department of Hepato-biliary and digestive surgery, CHU Estaing, Clermont-Ferrand, France); Julio Galindo (Department of General and Digestive Surgery, Hospital Universitario Ramón y Cajal, Madrid, Spain); Torben Glatz (Department of General and Visceral Surgery, Medical Center – University of Freiburg, Freiburg, Germany); Tarkan Jäger (Department of Surgery, Paracelsus Medical University, Salzburg, Austria); Maximilian Jarra (Department of General Surgery, Campus Virchow Klinikum, Charité, Universitätsmedizin Berlin, Berlin, Germany); Ninad Katdare (Department of General Oncology, Sir H. N. Reliance Foundation Hospital and Research Centre, Mumbai, India); Vahan Kepenekian (Department of Digestive Surgery, Centre Hospitalier Lyon Sud, Lyon, France); Vladimir M. Khomyakov (Department of thoracoabdominal cancer surgery, P.A. Hertsen Moscow Oncology Research Center, Moscow, Russia); Konstantinos Kothonidis (Department of Digestive Surgery, CHR Val de Sambre, Sambreville, Belgium); Nathalie Laplace (Department of Digestive Surgery, Centre Hospitalier Lyon Sud, Lyon, France); Vincent Lavoue (Department of Gynecology, CHU Rennes, Rennes, France); Kuno Lehmann (Department of Surgery and Transplantation, University Hospital of Zurich, Zurich, Switzerland); Craig Lynch (Division of Cancer Surgery, Peter MacCallum Cancer Centre, Melbourne, Victoria, Australia); Sanket Mehta (Department of Surgical Oncology, Saifee Hospital, Mumbai, India); Bogdan Moldovan (Department of General Surgery, “Sf. Constantin” Private Hospital Braşov, Romania); Aviram Nissan (Department of General and Oncological Surgery-Surgery C, The Chaim Sheba Medical Center, Tel Hashomer, Ramat Gan, Israel); Maciej Nowacki (Department of Surgical Oncology, Ludwik Rydygier’s Collegium Medicum, Nicolaus Copernicus University in Torun, Bydgoszcz, Poland); David Orry (Department of Surgical Oncology, Centre Georges-François Leclerc, Dijon, France); Gloria Ortega Pérez (Department of Surgical Oncology, MD Anderson, Madrid, Spain); Urs G. Pabst (Department of Surgery, Ruhr University Bochum, Bochum, Germany); Brice Paquette (Department of Digestive Surgery, CHU Jean Minjoz, Besançon, France); Marius Paskonis (Centre of Abdominal Surgery, Vilnius University Hospital Santariškių Klinikos, Vilnius, Lithuania); Pompiliu Piso (Department of General and Visceral Surgery, Barmherzige Brueder Hospital Regensburg, Regensburg, Germany); Marc Pocard (Department of Digestive and Visceral Surgery, APHP Lariboisière, Paris); Beate Rau (Department of Surgery, Campus Virchow-Klinikum and Charité Campus Mitte, Charité-Universitätsmedizin Berlin, Germany); Marc Reymond (Department of Surgery, University of Tübingen, Tübingen, Germany); Frederic Ris (Division of Digestive Surgery, University Hospitals of Geneva, Geneva, Switzerland); Manuela Robella (Unit of Surgical Oncology, Candiolo Cancer Institute-FPO, IRCCS, Turin, Italy); José Silvestre-Rodriguez (Department of General Surgery, Hospital Universitario de Gran Canaria Dr. Negrin, Las Palmas de Gran Canaria, Spain); Shivendra Singh (Department of Gastro-intestinal Oncosurgery & Liver Transplantation, Rajiv Gandhi Cancer Institute, New Delhi, India); S. P. Somashekhar (Department of Surgical Oncology, Manipal Comprehensive Cancer Center, Manipal Hospital, Bangalore, India); Claudio Soravia (Laparoscopic Robotic Surgery, Clinique Générale-Beaulieu, Geneva, Switzerland); Isabelle Sourrouille (Department of Surgical Oncology, Institut Gustave Roussy, Villejuif, France); Abelkader Taibi (Department of Digestive Surgery, CHU Dupuyren, Limoges, France); Clemens Tempfer (Department of Obstetrics and Gynecology, Ruhr-Universität Bochum, Bochum, Germany); Jared Torkington (Department of General Surgery, University Hospital of Wales, Cardiff, United Kingdom); Giuseppe Vizzielli (Division of Gynecologic Oncology, Catholic University of the Sacred Heart, Rome, Italy); Wouter Willaert (Department of GI Surgery and Cancer Research Institute Ghent [CRIG], Ghent University Hospital, Ghent, Belgium).
