# Supplementary material for: Consensus statement on safety measures for pressurized intraperitoneal aerosol chemotherapy
Source: Pleura Peritoneum. 2021 Nov 2;6(4):139–49. doi: 10.1515/pp-2021-0125 (PMC8719448; doi:10.1515/pp-2021-0125)
Supplement: Supplementary file 1 — Supplementary Material Details [file j_pp-2021-0125_suppl.docx]

**Appendix 1.** Delphi consensus on PIPAC safety: survey and recommendations

Consensus on safety measures for PIPAC

Pressurized IntraPeritoneal Aerosol Chemotherapy (PIPAC) delivers chemotherapeutic agents (CA) in aerosolized form to the patient, which causes potential occupational hazards for all healthcare providers present in the operating room (OR). Therefore, a stringent safety protocol has been proposed under two different validated versions [1, 2]. Subsequently, other studies have shown that with the implementation of safety measures, the risk of inhalation and surface contamination by CA is compatible with low risk of occupational hazard exposure [3-6].

However, a recent survey amongst PIPAC expert centers showed variable adherence to protective measures [7]. Another recent study on information relating to the risk of exposure suggested there is a need for continuous education [8].

Furthermore, knowledge concerning the risks of CA and the safety protocol was variable amongst OR professionals including surgeons, anesthetists, anesthesia nurses, scrub nurses and cleaning staff. Furthermore, institutional safety protocols also depend on national regulations.

**Objective**

The aim of this survey is to define consensus guidelines on safety measures for all OR members involved in PIPAC procedure to prevent the risk of occupational hazards and increase safety perception.

**Introduction**

PIPAC procedure involves a risk of exposure to liquid CA and to aerosolized CA. Both of these methods have dedicated preventive measures. These two aspects are discussed in two different chapters. The third chapter is dedicated to the importance of general aspects such as standardization, training, etc.

Exposure risks are different for the different members of the OR team, and protective measures might therefore also be different. For liquid CA, only the surgeon charging the injector is exposed to a larger volume of CA, while the rest of the surgical team deals with contaminated material at the end of the procedure. All other care providers only have minimal risk of exposure. Exposure risk for aerosolized CA is overall very low due to the existing safety protocols. Theoretically, only the staff entering the OR during the PIPAC phase (start of injection – exsufflation completed) is at high risk of exposure. Usually, this concerns 1-2 members of the surgical team.

Table 1 gives an overview on the type and estimated risk of exposure for the different care providers (typical situation) during the three different phases of the PIPAC procedure, namely before, during and after administration of CA. Highlighted are the situations with direct exposure (= high risk) as opposed to the situations with indirect exposure (= low risk).

In the following survey, the experts are asked to provide their level of agreement for, or against, the use of personal protective equipment (PPE) for the prevention of exposure to liquid or aerosolized CA (I), the use of environmental protection of the OR (II), the prevention of exposure to aerosolized CA (III), and their support for general safety precautions (IV). Personal protective measures will be studied separately for people with high (A) and low risk (B) of exposure.

**Table 1** Exposure risks for the different care providers during PIPAC

|  | Before PIPAC | PIPAC  administration - evacuation | After PIPAC | |
| --- | --- | --- | --- | --- |
| Exposure risk | Liquids | Aerosol | Liquids | Aerosol |
| Careproviders |  |  |  |  |
| Surgeon | +++ | +++ | ++ | + |
| Surgical team | + | (- or +++)* | ++ | + |
| Anesth. team | + | (- or +++)* | + | + |
| Circulators | + | - | + | + |
| Cleaning staff | - | - | + | + |

Surgeon: (un)loading of the injector, evacuation phase

Surgical team: surgical assistant, scrub nurse

Anesthesia team: doctor, nurse

Circulator, visitors, students, other

Cleaning staff

+++ direct exposure to high concentrations of liquid/aerosolized (hypothetical) CA

++ direct exposure to low concentrations of liquid CA

+ no direct exposure to liquid CA or low (hypothetical) exposure to aerosol CA

- outside the room = no risk

* high risk of exposure only in the (very unlikely) emergency situation

1. **Personal protective equipment for the prevention of exposure to liquid or aerosolized CA**

CA used in PIPAC are Platin compounds (oxaliplatin and cisplatin) and doxorubicin [2]. These CA are classified as toxic agents and are recognized as being a risk for healthcare workers [9]. Other drugs are currently in the development phase.

For both Hyperthermic IntraPeritoneal Chemotherapy (HIPEC) and PIPAC procedures, the handling of CA represents additional risk of exposure for the team members of the OR. An additional risk, specific to PIPAC, is the exposure to aerosol droplets caused by potential sealing defects during CA administration. Exposure to CA during PIPAC can occur through inhalation of aerosols or vapors and through direct contact (dermal or ocular) with contaminated surfaces or materials [2]. A number of studies have investigated OR air contamination with cisplatin by measuring its concentration in air samples [2, 4, 6] and surface samples (gloves, hands, devices, injector, trocars and floor) [2, 5, 6]. These studies reported no (or minimal) contamination. Another study [3] investigated the concentration of oxaliplatin in surface samples, and found no contamination. Other studies monitored healthcare workers with blood samples or urines samples [4, 5], and found no contamination. Currently, there is no strong consensus about PPE required for handling CA or for preventing exposure during PIPAC procedures. Also, differences in current practices for prevention have been observed among expert centers [7]. In 2016, safety guidelines for administration of HIPEC were published [10]. The following paragraph summarizes current measures for PPE during HIPEC procedures: gowns should be watertight and with closed fronts, long sleeves, and elastic closed cuffs. Only gowns with polyethylene or vinyl coatings provide adequate protection against dermal exposure to CA [10]. Shoes should be closed and the use of overshoes is recommended only in the direct working area [10]. Use of gloves should meet the American Society for Testing and Materials (ASTM) standard or fulfill the EN 374 specifications. Powdered gloves should be avoided. Double gloving confers a higher protection, as the air layer between the two gloves is an effective barrier. According to manufacturers, gloves should be changed every 30 minutes [10]. Plastic goggles should be worn to prevent ocular exposure; alternatively a safety shield may be used. Normal optical glasses shouldn’t be considered as PPE, as they do not provide sufficient ocular protection [10, 11]. Volume, concentrations and handling of liquid CA is quite limited during PIPAC, and it is unclear whether similar protection should be used by extrapolation from HIPEC [10]. Filtering face piece (FFP) mask class 2 (filtering 94% of particles) should be carried by non-operating members, and FFP mask class 3 (filtering 99% of particles, from 0,01 to 1 μm) carried by operating members (surgeons, scrub nurse) to avoid direct exposure or inhalation of aerosols (oxaliplatin, cisplatin and doxorubicin) according to European Norms 149 [12], and should be changed every two hours [11].

The following 9 recommendations concern personal protection against exposure to liquid and aerosolized CA for both, care providers at high risk (A) and at low risk (B) of exposure:

In order to prevent/limit exposure to liquid chemotherapy agents during PIPAC …

1. … the use of a reinforced surgical gown with watertight material instead of a standard surgical gown …
2. … the use of plastic overshoes/shoe covers instead of no covers …
3. … the use of routine double gloving instead of wearing a single pair of surgical gloves …
4. … the use of an inner pair of (unpowdered) gloves resistant to CA and doubled with an outer pair instead of a double pair of standard gloves …
5. … change of gloves every 30 minutes instead of keeping the same gloves from the beginning to the end of the procedure …
6. … the use of additional long-sleeve cuffs instead of no additional protection of the junction between gloves and gown …
7. … any kind of eye protection by wearing at least normal optical glasses instead of no protective eyewear …
8. … the use of plastic goggles or protective shields (=specific protective eyewear) instead of standard protective measures (above) …
9. … the use of FFP-3 masks instead of standard surgical or FFP-2 masks …

A … for care providers with high risk of exposure …

| A | should be indicated routinely. |
| --- | --- |
| B | could be indicated. |
| C | should not be indicated. |
| D | is not indicated. |

B … for care providers with low risk of exposure …

| A | should be indicated routinely. |
| --- | --- |
| B | could be indicated. |
| C | should not be indicated. |
| D | is not indicated. |

**Results summary and recommendations:**

- 1. *For caregivers with* ***high risk*** *of exposure*

*Reinforced gown*

The wearing of reinforced gown with watertight material instead of a standard surgical gown **should be indicated** **routinely** to prevent exposure to CA during PIPAC.

*Recommendation strength*: Strong positive (85.1% agreement, consensus reached).

*Shoe covers*

The wearing of shoe covers instead of no covers **should be indicated** **routinely** to prevent exposure to CA during PIPAC.

*Recommendation strength*: Strong positive (72.3% agreement, consensus reached).

*Double gloving*

The wearing of double gloving instead of wearing a single pair **should be indicated** **routinely** to prevent exposure to CA during PIPAC.

*Recommendation strength*: Strong positive (87.2% agreement, consensus reached).

*Inner pair of gloves resistant to CA*

The wearing of an inner pair of gloves resistant to CA and doubled with an outer pair instead of a double pair of standard gloves **should be indicated** **routinely** to prevent exposure to CA during PIPAC.

*Recommendation strength*: Strong positive (51.1% agreement, consensus reached).

*Change of gloves every 30 minutes*

**No consensus** was reached on the changing of gloves every 30 minutes instead of keeping the same gloves from the beginning to the end of the procedure to prevent exposure to CA during PIPAC.

*Recommendation strength*: No consensus.

*Long-sleeve cuffs*

The wearing of additional long-sleeve cuffs instead of no additional protection of the junction between gloves and gown **should not be indicated** to prevent exposure to CA during PIPAC.

*Recommendation strength*: Weak negative (55.3% agreement, consensus reached).

*Minimal protective eyewear*

The wearing of any kind of eye protection with at least normal optical glasses **should be indicated** **routinely** to prevent exposure to CA during PIPAC.

*Recommendation strength*: Strong positive (87.2% agreement, consensus reached).

*Specific protective eyewear*

The wearing of plastic goggles or protective shields **should be indicated** **routinely** to prevent exposure to CA during PIPAC.

*Recommendation strength*: Strong positive (59.6% agreement, consensus reached).

*FFP mask class 3*

The wearing of FFP mask class 3 instead of standard or FFP mask class 2 **should be indicated** **routinely** to prevent exposure to aerosolized CA during PIPAC.

*Recommendation strength*: Strong positive (53.2% agreement, consensus reached).

- 1. *For care providers with* ***low risk*** *of exposure*

*Reinforced gown*

The wearing of reinforced gown with watertight material instead of a standard surgical gown **could be indicated** to prevent exposure to CA during PIPAC.

*Recommendation strength*: Weak positive (55.3% agreement, consensus reached).

*Shoe covers*

The wearing of shoe covers or overshoes instead of no covers **could be indicated** to prevent exposure to CA during PIPAC.

*Recommendation strength*: Weak positive (66% agreement, consensus reached).

*Double gloving*

The wearing of double gloving instead of wearing a single pair **could be indicated** to prevent exposure to CA during PIPAC.

*Recommendation strength*: Weak positive (61.7% agreement, consensus reached).

*Inner pair of gloves resistant to CA*

The wearing of an inner pair of gloves resistant to CA and doubled with an outer pair instead of a double pair of standard gloves **could be indicated** to prevent exposure to CA during PIPAC.

*Recommendation strength*: Weak positive (51.1% agreement, consensus reached).

*Change of gloves every 30 minutes*

The change of gloves every 30 minutes instead of keeping the same gloves from the beginning to the end of the procedure **is not indicated** to prevent exposure to CA during PIPAC.

*Recommendation strength:* Strong negative (57.4% agreement, consensus reached).

*Long-sleeve cuffs*

The wearing of additional long-sleeve cuffs instead of no additional protection of the junction between gloves and gown **is not indicated** to prevent exposure to CA during PIPAC.

*Recommendation strength*: Strong negative (66% agreement, consensus reached).

*Minimal protective eyewear*

*Summary and recommendation:* the wearing of any kind of eye protection with at least normal optical glasses **could be indicated** to prevent exposure to CA during PIPAC.

*Recommendation strength*: Weak positive (59.6% agreement, consensus reached).

*Specific protective eyewear*

The wearing of plastic goggles or protective shields **could be indicated** to prevent exposure to CA during PIPAC.

*Recommendation strength:* Weak positive (61.7% agreement, consensus reached).

*FFP mask class 3*

**No consensus** was reached on the wearing of FFP mask class 3 instead of standard or FFP mask class 2 to prevent exposure to aerosolized CA during PIPAC.

*Recommendation strength*: No consensus.

1. **Environmental protection**

In order to prevent surface contamination, absorbent mats are placed on the floor around the injector and operating during HIPEC procedures [10]. In PIPAC, one study demonstrated that the contamination of the floor was lower when an absorbent mat was disposed near the injector [3]. The empirical PIPAC protocol recommends positioning a specific waste bin under the injector head and covering the high-pressure line by with transparent plastic in order to contain leakage of liquid CA in case of disconnection between the syringe and high-pressure line [13]. No data is available to support or refute this practice. One multi-center study [3] demonstrated a decrease of local contamination when surgical disposables were removed “en bloc”, as compared to removal after disconnection. The German protocol [2] suggests disposal of (potentially) contaminated material such as tissues, tubes, lines, trocars, aerosolizer, and other devices such as drapes and sponges, and all PPE described above into special, sealed and labeled containers. As for the instruments used during PIPAC procedure (biopsy forceps, laparoscopic scissors and laparoscopic camera), only the laparoscopic camera is exposed to CA. One study [14] demonstrated minimal traces of platinum on the camera directly after PIPAC, but none after the sterilization process. This shows that laparoscopic cameras can be safely reused after PIPAC. Disposable covers on the injector monitor during PIPAC could help prevent the transfer of potential contamination to other surfaces [3].

The following 7 recommendations concern environmental protection against exposure to liquid CA:

In order to prevent/limit exposure to liquid chemotherapy agents during PIPAC …

1. … the use of absorbent mats on the floor next to the injector and operative table instead of no covering of the potentially exposed floor …
2. … placing a labeled container under the injector head to contain spillage in case of accidental disconnection (syringe-high pressure line) instead of no preventive measure …
3. … wrapping a transparent cover sheet around the high-pressure line to contain spillage in case of accidental disconnection (syringe-high pressure line) instead of no preventive measure …
4. … the en-bloc removal of contaminated disposables into the labeled containers instead of disconnection …
5. … the use of dedicated, sealed and labeled chemotherapy waste containers instead of standard waste containers …
6. … the use of low-price single-use laparoscopic cameras instead of re-using sterilized laparoscopic cameras …
7. …the use of disposable covers on the injector monitor instead of no protective cover …

| A | should be indicated routinely. |
| --- | --- |
| B | could be indicated. |
| C | should not be indicated. |
| D | is not indicated. |

**Results summary and recommendations:**

*Absorbent mats*

The use of absorbent mats on the floor next to the injector and operative table instead of no covering of the potentially exposed floor **should be indicated** **routinely** to prevent exposure to liquid CA during PIPAC.

*Recommendation strength*: Strong positive (95.7% agreement, consensus reached).

*Labeled container under the injector head*

Placing a labeled container under the injector head to contain spillage in case of accidental disconnection (syringe-high pressure line) instead of no preventive measure **should be indicated** **routinely** to prevent exposure to liquid CA during PIPAC.

*Recommendation strength*: Strong positive (95.7% agreement, consensus reached).

*Transparent cover sheet*

Wrapping a transparent cover sheet around the high-pressure line to contain spillage in case of accidental disconnection (syringe-high pressure line) instead of no preventive measure **should be indicated** **routinely** to prevent exposure to liquid CA during PIPAC.

*Recommendation strength*: Strong positive (89.4% agreement, consensus reached).

*En-bloc removal*

The en-bloc removal of contaminated disposables into the labeled containers instead of disconnection **should be indicated** **routinely** to prevent exposure to liquid CA during PIPAC.

*Recommendation strength*: Strong positive (93.6% agreement, consensus reached).

*Waste containers*

The use of dedicated, sealed and labeled chemotherapy waste containers instead of standard waste containers **should be indicated** **routinely** to prevent exposure to liquid CA during PIPAC.

*Recommendation strength*: Strong positive (97.9% agreement, consensus reached).

*Single-use laparoscopic cameras*

*Summary and recommendation:* the use of low-price single-use laparoscopic cameras instead reusing sterilized laparoscopic cameras **is not indicated** to prevent exposure to liquid CA during PIPAC.

*Recommendation strength*: Strong negative (93.6% agreement, consensus reached).

*Disposable covers on the injector monitor*

The use of disposable covers on the injector monitor instead of no protective cover **could be indicated** to prevent exposure to liquid CA during PIPAC.

*Recommendation strength*: Weak positive (72.3% agreement, consensus reached).

1. **Prevention of exposure to aerosolized CA**

The pioneer PIPAC team in Germany developed a safety protocol which includes 3 levels of containment. It was initially designed as an empirical safety protocol [15], and first tested in Germany [2], and detected no cisplatin in the air. These results were later validated by two studies from two different centers [4, 6].

The tightness of the abdomen, the first level, is controlled by a “zero-flow” (max 0.4 L/min) from the CO_2_ insufflator, and systemic use of balloon trocars [16].

The second level of containment consists of the OR, the ventilation system, and the intrinsic dilution capacity. Initially, PIPAC could be safely performed, as long as the OR disposes of a laminar air flow system (Isonorm 14644) [16]. PIPAC was validated without mandatory laminar airflow in the French protocol given the set of modifications brought to the initial German protocol [1]**.** The main feature was a transparent plastic cover protection connected to a smoke filtration device as an alternative to laminar airflow. Air measurements were negative for cisplatin and doxorubicin [17].

The third level consists of remote administration and controlling of the procedure. After the video-optic in the 5-mm trocar is focused to the nozzle orifice, the entire staff leaves the OR for remote application of CA. Anesthesia and surgical teams position themselves in the lock room with monitor view for the anesthesist and the surgeon (laparoscopy video monitor and the high-pressure injector) to check for correct aerosolization [13]. At the end of the PIPAC procedure, the toxic aerosol is evacuated through a Closed Aerosol Waste System (CAWS). CAWS consists of a closed line connecting the valve of a trocar to the suction plug of the wall, routinely used by the anesthesiologists to evacuate narcotic gases. Two micro-particle filters are placed one after the other to limit the risk of contamination of the environment. According to the pioneer German team, closing CAWS with two valves is recommended to prevent any inadvertent exsufflation of the pneumoperitoneum [16]. In one study, no platinum (cisplatin and oxaliplatin) was detected on the filters nor in the air-seal container liquid composing the CAWS [6]. In the french protocol , the remaining toxic aerosol is evacuated over a closed surgical smoke evacuation system as described by Cazauran et al [1].

The following 8 recommendations concern protection against exposure to aerosolized CA:

In order to prevent/limit exposure (inhalation) to aerosolized chemotherapy agents during PIPAC …

1. … use of single-use trocars with balloon and extra-abdominal obturators instead of standard trocars …
2. … control of an air-tight pneumoperitoneum (“zero-flow” 0.0-0.4 L/min) before start of the CA aerosolization instead of no control …
3. … activation of an advanced OR ventilation system instead of a standard or even no specific ventilation system …
4. … activation of laminar air flow instead of an advanced OR ventilation system …
5. … the additional use of a transparent plastic cover protection connected to a smoke filtration device (“French system”) instead of no laminar airflow …
6. … remote administration of CA with all OR team members leaving the OR instead of possible presence of OR team members in the OR …
7. … remote video monitoring (fixed camera, outside screen) instead of “blind administration” …
8. … evacuation of the toxic aerosol via a closed system (CAWS) including microparticle filters / surgical smoke evacuation system (French protocol) instead of a standard laparoscopic suction device or open exsufflation …

| A | should be indicated routinely. |
| --- | --- |
| B | could be indicated. |
| C | should not be indicated. |
| D | is not indicated. |

**Results summary and recommendations:**

*Single-use trocars with balloon*

The use of single-use trocars with balloon and extra-abdominal obturators instead of standard trocars **should be indicated** **routinely** to prevent exposure to aerosolized CA during PIPAC.

*Recommendation strength*: Strong positive (93.6% agreement, consensus reached).

*Airtight pneumoperitoneum*

The control of an airtight pneumoperitoneum (“zero-flow” 0.0-0.4 L/min) before start of the CA aerosolization instead of no control **should be indicated** **routinely** to prevent exposure to aerosolized CA during PIPAC.

*Recommendation strength*: Strong positive (100% agreement, consensus reached).

*Advanced OR ventilation system*

The activation of an advanced OR ventilation system instead of a standard or even no specific ventilation system **should be indicated** **routinely** to prevent exposure to aerosolized CA during PIPAC.

*Recommendation strength*: Strong positive (91.5% agreement, consensus reached).

*Laminar air flow*

The activation of laminar air flow instead of an advanced OR ventilation system **could be indicated** to prevent exposure to aerosolized CA during PIPAC.

*Recommendation strength*: Positive (48.9% weak positive agreement, consensus reached with >70% agreement for combined weak and strong recommendation).

*Plastic cover protection (“French system”)*

The additional use of a transparent plastic cover protection connected to a smoke filtration device (also called “French system”) instead of no laminar air flow **could be indicated** to prevent exposure to aerosolized CA during PIPAC.

*Recommendation strength*: Weak positive (55.3% agreement, consensus reached).

*Remote administration*

Remote administration of CA with all OR team members leaving the OR instead of possible presence of OR team members in the OR **should be indicated** **routinely** to prevent exposure to aerosolized CA during PIPAC.

*Recommendation strength*: Strong positive (95.7% agreement, consensus reached).

*Remote video monitoring*

Remote video monitoring (fixed camera and outside screen) instead of “blind administration” **should be indicated** **routinely** to prevent exposure to aerosolized CA during PIPAC.

*Recommendation strength*: Strong positive (89.4% agreement, consensus reached).

*Toxic aerosol evacuation*

Toxic aerosol evacuation via a Closed Aerosol Waste System including micro-particle filters or via a surgical smoke evacuation system (“French system”) instead of a standard laparoscopic suction device or open exsufflation **should be indicated** **routinely** to prevent exposure to aerosolized CA during PIPAC.

*Recommendation strength*: Strong positive (97.9% agreement, consensus reached).

1. **General preventive measures**

Appropriate labeling and restricted access to the OR during PIPAC procedures are currently performed in most centers. This was extrapolated from recommended practices during HIPEC procedures, and based on common sense [10]. In analogy to the WHO surgical safety checklist, and taking into account the occupational risk of CA administration during PIPAC, an extended and dedicated PIPAC safety checklist (4-eyes validation principle) was established by the German pioneer group. It was then adopted by other centers and advocated in ISSPP training modules [18]. Emergency kits, including absorbent devices, mild soap, bleach and eye wash kit, provide quick absorption of HIPEC solution and first aid [19]. It has been suggested without experimental grounds that pregnant women should not take part in HIPEC procedures [10, 19], and this is also advocated for PIPAC procedures. There is no particular mention in the literature relating to students or medical visitors. In HIPEC procedure, the importance of effective cleaning methods was demonstrated [20] after detecting platinum contamination on the floor of the OR, up to 3 days after the procedure. In the PIPAC procedure, injector contamination seems to reveal insufficient cleaning methods. In one study [3], contamination was higher before PIPAC as compared to after PIPAC, while another study [5] showed that contamination remained after cleaning. These results might motivate a control of the efficiency of the cleaning procedure or of an extended cleaning procedure of the injector to avoid recurrent contamination in the OR and on PIPAC reusable devices. A revised cleaning method with triple-wiping of the PIPAC device was reported effective in reducing injector contamination in the follow-up of one study, but without providing data [5]. A survey of the French centers involved in HIPEC procedures showed that waste management of the equipment in direct contact with liquid CA enters a special circuit for “cytotoxic waste” with incineration at 1200^0^ C [19]. There is currently no specific data for PIPAC. A recent survey amongst PIPAC expert centers showed a rather low rate of adhesion to protection measures. Safety awareness did not reach expected levels for a highly standardized procedure, with a lower information score among anesthesiologists and cleaning staff. Availability of emergency kits in case of accidental exposure was unknown for 50% of responders. Most OR team members seek supplementary information about the risks related to CA administration [7]. Another recent study demonstrated that non-medical caregivers in the OR are aware of the occupational hazards related to the use of CA. However, there is a high need of continuous education for the involved personnel [8].

The following 10 recommendations concern general preventive measures:

1. During PIPAC procedure, labeled entrance of the OR instead of standard OR policy …
2. During PIPAC procedure, restricted access for all personnel not directly involved (including students or visitors, for instance) instead of standard OR policy …
3. During PIPAC procedure, the use of a checklist before aerosolization to double-check all mandatory safety components …
4. During PIPAC procedure, the availability of an emergency kit for accidental exposure to chemotherapeutic agents, and ready to use in the OR …
5. Extended injector cleaning procedure (triple wiping of the PIPAC injector) to reduce risk of recurrent OR contamination, compared to standard cleaning procedure…

| A | should be indicated routinely. |
| --- | --- |
| B | could be indicated. |
| C | should not be indicated. |
| D | is not indicated. |

1. Access to PIPAC procedure for pregnant collaborators …

| A | should be permitted routinely. |
| --- | --- |
| B | could be permitted. |
| C | should not be permitted. |
| D | is not permitted. |

1. Prior information related to potential biohazards of administration of CA by a dedicated training for the following professional groups involved in PIPAC…
2. Prior training on safety measures for the following professional groups involved in PIPAC …
3. Prior information on emergency measures required in case of accidental exposure to chemotherapeutic agents by a dedicated training for the following professional groups involved in PIPAC …
4. Prior information on waste management and cleaning procedures for the following professional groups involved in PIPAC …

|  |  | Surgeons | Scrub nurses | Anesthesia team | Cleaning staff |
| --- | --- | --- | --- | --- | --- |
| A | should be indicated routinely. |  |  |  |  |
| B | could be indicated. |  |  |  |  |
| C | should not be indicated. |  |  |  |  |
| D | is not indicated. |  |  |  |  |

**Results summary and recommendations:**

*Labeled entrance*

Labeled entrance of the OR instead of standard OR policy **should be indicated** **routinely** to prevent exposure to CA during PIPAC.

*Recommendation strength*: Strong positive (95.7% agreement, consensus reached).

*Restricted access*

Restricted access for all personnel directly involved (including students or visitors, for instance) instead of standard OR policy **should be indicated** **routinely** to prevent exposure to CA during PIPAC.

*Recommendation strength*: Strong positive (89.4% agreement, consensus reached).

*Checklist before aerosolization*

The use of a checklist before aerosolization to double-check all mandatory safety components **should be indicated** **routinely** to prevent exposure to CA during PIPAC.

*Recommendation strength*: Strong positive (100% agreement, consensus reached).

*Emergency kit*

The availability of an emergency kit for accidental exposure to CA, and ready to use in the OR **should be indicated** **routinely** during PIPAC.

*Recommendation strength*: Strong positive (95.7% agreement, consensus reached).

*Extended injector cleaning procedure*

Extended injector cleaning procedure (triple wiping of the injector) instead of standard cleaning procedure **could be indicated** to reduce risk of recurrent OR contamination after PIPAC.

*Recommendation strength*: Weak positive (51.1% agreement, consensus reached).

*Pregnant collaborators*

*A*ccess for pregnant collaborators **should not be permitted** during PIPAC.

*Recommendation strength*: Strong negative (55.3% agreement, consensus reached).

Concerning the four items related to *prior information and training on safety measures*, the panelists consensually delivered strong positive recommendations for the 4 items and professional groups, respectively surgeons, scrub nurses, anesthesia team and cleaning staff.

**Results summary and recommendations:**

*Prior information on related biohazards of CA administration, emergency measures, waste management, cleaning procedures as well as training on safety on safety measures* ***should be routinely given*** *to the entire OR team (surgeons, scrub nurses, anesthesia team and cleaning staff) regularly involved in PIPAC procedures.*

References

1. Cazauran, J.B., et al., Pressurized Intraperitoneal Aerosol Chemotherapy (PIPAC) Procedure for Non-resectable Peritoneal Carcinomatosis (with Video). J Gastrointest Surg, 2018. 22(2): p. 374-375.

2. Solass, W., et al., Pressurized intraperitoneal aerosol chemotherapy (PIPAC): occupational health and safety aspects. Ann Surg Oncol, 2013. 20(11): p. 3504-11.

3. Ametsbichler, P., et al., Occupational exposure to cisplatin/oxaliplatin during Pressurized Intraperitoneal Aerosol Chemotherapy (PIPAC)? Eur J Surg Oncol, 2018. 44(11): p. 1793-1799.

4. Graversen, M., P.B. Pedersen, and M.B. Mortensen, Environmental safety during the administration of Pressurized IntraPeritoneal Aerosol Chemotherapy (PIPAC). Pleura Peritoneum, 2016. 1(4): p. 203-208.

5. Ndaw, S., et al., Occupational exposure to platinum drugs during intraperitoneal chemotherapy. Biomonitoring and surface contamination. Toxicol Lett, 2018. 298: p. 171-176.

6. Willaert, W., P. Sessink, and W. Ceelen, Occupational safety of pressurized intraperitoneal aerosol chemotherapy (PIPAC). Pleura Peritoneum, 2017. 2(3): p. 121-128.

7. Clerc, D.H., M.; Sgarbura, O., Current practice and perceptions of safety protocols for the use of intraperitoneal in the operating room: Results of the IP-OR international survey. Presented at 1st ISSPP International congress, unpublished data 2020, 2020.

8. Al Hosni, M., et al., Non-medical caregivers and the use of intraperitoneal chemotherapy in the operating theatre: A survey on the perception of safety. J Visc Surg, 2020.

9. Preventing occupational exposures to antineoplastic and other hazardous drugs in health care settings. NIOSH Publication, 2004.

10. Kyriazanos, I., et al., Operating personnel safety during the administration of Hyperthermic Intraperitoneal Chemotherapy (HIPEC). Surg Oncol, 2016. 25(3): p. 308-14.

11. Simon, L., et al., Hyperthermic intraoperative intraperitoneal chemotherapy (HIPEC): evaluation, prevention and policies to avoid occupational exposure for operating room personnel. Bull Cancer, 2009. 96(10): p. 971-7.

12. Alyami, M., et al., Pressurised intraperitoneal aerosol chemotherapy: rationale, evidence, and potential indications. Lancet Oncol, 2019. 20(7): p. e368-e377.

13. Giger-Pabst, U. and C.B. Tempfer, How to Perform Safe and Technically Optimized Pressurized Intraperitoneal Aerosol Chemotherapy (PIPAC): Experience After a Consecutive Series of 1200 Procedures. J Gastrointest Surg, 2018. 22(12): p. 2187-2193.

14. Demtroder, C.e.a., Platinum contamination of laparoscopic instruments during pressurized intra peritoneal aerosol chemotherapy (PIPAC). Journal of Pharmacy and Pharmaceutical Sciences, 2016.

15. Reymond, M.A., Solass, W., PIPAC Pressurized intraperitoneal aerosol chemotherapy - Cancer under pressure. 2014: De Gruyter.

16. Reymond, L.e.a., Pressurized intraperitoneal aerosol chemotherapy (PIPAC): occupational health and safety management.

17. Delhorme, J.B., et al., Occupational safety of pressurized intraperitoneal aerosol chemotherapy (PIPAC) in an operating room without laminar airflow. J Visc Surg, 2019. 156(6): p. 485-488.

18. Hubner, M., et al., Pressurized IntraPeritoneal Aerosol Chemotherapy - Practical aspects. Eur J Surg Oncol, 2017. 43(6): p. 1102-1109.

19. Ferron, G., et al., Professional risks when carrying out cytoreductive surgery for peritoneal malignancy with hyperthermic intraperitoneal chemotherapy (HIPEC): A French multicentric survey. Eur J Surg Oncol, 2015. 41(10): p. 1361-7.

20. Schierl, R., et al., Low surface contamination by cis/oxaliplatin during hyperthermic intraperitoneal chemotherapy (HIPEC). Eur J Surg Oncol, 2012. 38(1): p. 88-94.
